# A Lightweight Temporal Convolutional Network for Contactless SPPB-Aligned Functional Fall-Risk Stratification in Older Adults Using Monocular RGB Video

**DOI:** 10.3390/s26123894

**Published:** 2026-06-18

**Authors:** Kai-Chih Lin, Rong-Jong Wai, Hung-Yu Chang Chien

**Affiliations:** 1 Department of Electronic and Computer Engineering, National Taiwan University of Science and Technology, Taipei 106, Taiwan; d10602201@mail.ntust.edu.tw; 2 Department of Health Promotion and Health Education, National Taiwan Normal University, Taipei 106, Taiwan; 81205007e@ntnu.edu.tw

**Keywords:** fall-risk assessment, functional risk stratification, contactless sensing, monocular RGB video, human pose estimation, temporal convolutional network, skeletal biomechanics, edge AI, explainable artificial intelligence

## Abstract

Falls among older adults remain a major public health concern, yet scalable and interpretable sensing approaches for functional fall-risk stratification remain limited. This study presents a lightweight contactless framework for five-level Short Physical Performance Battery (SPPB)-aligned functional fall-risk stratification using monocular RGB video. A total of 688 community-dwelling older adults completed SPPB-aligned assessments, including balance, five-times sit-to-stand, and 3 m gait tasks. Because prospective fall-event outcomes were unavailable, supervised labels were constructed from a pre-specified SPPB-aligned functional risk index rather than observed future falls. BlazePose-based two-dimensional keypoints were extracted, normalized using pelvis-centered and height-scaled transformations, and represented as temporal skeletal trajectories. Biomechanical descriptors were fused with embeddings from the proposed Temporal Convolutional Artificial Intelligence Fall-Risk Network (TCAI-FallNet). Participant-level data partitioning was used to reduce data leakage. TCAI-FallNet achieved a macro-averaged area under the curve of 0.91 and an overall accuracy of 81.3%. The trained model had a footprint under 3 MB, and TCN inference latency was below 20 ms per sequence under workstation-based evaluation. These findings suggest that TCAI-FallNet may support contactless SPPB-aligned functional mobility risk stratification, while prospective fall-event validation remains necessary.

## 1. Introduction

Falls are among the most serious health and safety concerns affecting older adults, often leading to injury, functional decline, loss of independence, institutionalization, and increased healthcare expenditure. The World Health Organization has identified falls as a major public health issue in aging populations, emphasizing the need for effective prevention and early risk identification strategies [1]. Because fall risk is multifactorial and can be influenced by balance control, gait regularity, lower-limb strength, and postural stability, reliable assessment tools are essential for community-based screening and timely intervention. Early clinical efforts to identify fall-prone individuals relied primarily on observational scales and structured assessments, which provided useful screening information but were limited by subjectivity, evaluator variability, and insufficient temporal resolution for capturing subtle movement deterioration [2].

Recent advances in computer vision and human pose estimation have created new opportunities for noncontact movement assessment. Markerless pose estimation methods can extract skeletal keypoints from ordinary video recordings without requiring wearable sensors or body-attached markers. OpenPose demonstrated the feasibility of real-time multi-person two-dimensional pose estimation using part affinity fields [3], while high-resolution networks further improved pose localization accuracy through enhanced spatial representation learning [4]. For practical deployment, lightweight pose estimation frameworks such as BlazePose provide an efficient alternative for on-device body tracking and real-time skeletal landmark extraction [5]. These developments suggest that RGB video can serve as a scalable sensing modality for movement analysis in older adults, particularly in community and home environments where wearable compliance and specialized laboratory equipment may be difficult to maintain.

Despite these advances, translating camera-based pose estimation into clinically meaningful fall-risk assessment remains challenging. Standardized functional assessment frameworks, such as the Short Physical Performance Battery (SPPB), evaluate lower-extremity function through balance, gait speed, and sit-to-stand performance, and have been widely associated with disability, mobility decline, and adverse health outcomes in older adults [6]. Gait variability has also been shown to be strongly associated with fall risk in community-living older adults, indicating that temporal irregularities in movement contain important information about postural control and functional decline [7]. However, many vision-based or sensor-based fall-related systems have focused on detecting fall events, abnormal postures, or binary risk outcomes rather than characterizing graded deterioration in dynamic postural control.

Existing sensing studies have provided important foundations for fall detection and posture analysis. Camera-based systems using OpenPose and multiview information have shown robust performance in abnormal human-posture recognition [8]. Wearable inertial measurement unit (IMU)-based approaches have also demonstrated promising results for fall detection using deep learning models [9], and pre-impact fall detection systems based on accelerometers have been explored for injury prevention [10]. Nevertheless, wearable systems often require correct sensor placement, regular usage, battery maintenance, and long-term user compliance, which may limit their scalability among older adults. In contrast, computer vision approaches can reduce user burden by enabling contactless assessment, but their clinical interpretability and robustness remain dependent on appropriate feature extraction, temporal modeling, and alignment with established functional assessment paradigms.

Several recent studies have begun to bridge this gap by using computer vision and machine learning to assess gait, mobility, and fall risk. Vision-based gait variable measurement has been applied to assess mobility and fall risk in older adults with dementia [11], while machine-learning approaches using gait-cycle information have shown potential for fall-risk classification in older populations [12]. However, many existing models rely on isolated gait features or short temporal windows, which may not fully capture the integrated movement characteristics expressed across balance, sit-to-stand, and walking tasks. Since functional mobility decline develops through changes in both static stability and dynamic movement transitions, a unified temporal representation across multiple assessment tasks may provide a more informative basis for fall-risk stratification.

Temporal sequence modeling is therefore critical for extracting meaningful patterns from skeletal movement data. Temporal Convolutional Networks (TCNs) have demonstrated strong capability in modeling sequential data through causal and dilated convolutions, offering stable temporal representation and computational efficiency compared with recurrent architectures [13]. Attention-based Transformer models can also capture long-range dependencies, but they generally require larger datasets and higher computational resources, which may reduce suitability for lightweight sensing applications [14]. For real-world fall-risk screening, an ideal model should simultaneously provide accurate classification, low inference latency, compact deployment size, and interpretable links between model predictions and biomechanical movement characteristics.

Recent *Sensors* studies further support the relevance of vision-based and markerless sensing for older adult mobility assessment. Multimodal sensor-based mobility assessment has integrated PoseNet-derived gait dynamics with body composition indicators for older adults [15]. Markerless motion capture parameters associated with fall risk and frailty have also been systematically reviewed, highlighting the potential of noncontact skeletal motion analysis for geriatric assessment [16]. In addition, vision-based sensor systems for human gait analysis have been comprehensively reviewed, reinforcing the importance of camera-based sensing for scalable movement evaluation [17]. These studies indicate that markerless visual sensing is increasingly recognized as a practical direction for mobility and fall-risk assessment, but further work is needed to develop interpretable, efficient, and clinically aligned models for multi-level fall-risk classification.

To address these challenges, this study proposes TCAI-FallNet, a lightweight temporal convolutional model for contactless SPPB-aligned functional fall-risk stratification in older adults. The proposed framework uses monocular RGB video to extract skeletal trajectories, applies pelvis-centered and height-normalized transformations, and fuses interpretable biomechanical descriptors with TCN-derived temporal embeddings. Unlike acute fall-event detection systems, the proposed approach focuses on graded functional mobility risk stratification derived from SPPB-aligned assessment tasks and vision-based skeletal features.

The main contributions of this study are as follows. First, a contactless RGB video-based sensing framework is developed for SPPB-aligned mobility assessment in community-dwelling older adults. Second, biomechanical descriptors, including mediolateral center-of-mass sway, sit-to-stand temporal efficiency, hip–knee angular asymmetry, and stride-time variability, are integrated with deep temporal embeddings to enhance both predictive performance and interpretability. Third, a lightweight dilated TCN architecture is designed to capture long-range postural dependencies while maintaining low model-level inference latency and a compact computational footprint that may support future edge-oriented implementation. Finally, SHAP-based feature attribution is used to examine the contribution of biomechanical and learned temporal features, providing interpretable evidence for SPPB-aligned functional risk classification decisions.

## 2. Materials and Methods

### 2.1. Study Participants and Ethical Approval

This study recruited 688 community-dwelling older adults who participated in a standardized functional mobility assessment in Taiwan. Eligible participants were aged 65 years or older and were able to complete the required mobility tasks, including standing balance, five-times sit-to-stand, and a 3 m usual-pace walking test. The participant age range was 65–92 years, and 64.2% of the cohort were female. The present cohort was independent from the population recruited in our previous AI-assisted intervention study. The current analysis focused specifically on validating the signal-processing architecture and classification performance of the proposed TCAI-FallNet framework.

All assessments were conducted under standardized community-based screening conditions. The study was approved by the Institutional Review Board of En Chu Kong Hospital (ECKIRB1071204). All participants were informed of the study objectives, assessment procedures, and data usage before enrollment, and written informed consent was obtained from all participants.

### 2.2. Functional Mobility Assessment Protocol

The experimental protocol followed the Short Physical Performance Battery (SPPB), which evaluates lower-extremity function through balance, chair-stand performance, and gait speed. These components correspond to clinically relevant domains of fall-risk assessment, including static postural control, lower-limb strength, transitional movement ability, and walking function. Sensor-assisted functional mobility assessment systems, such as environment-adaptive Timed Up and Go testing using wearable inertial sensors, have demonstrated the value of objective movement measurement for fall-risk assessment [18]. Building on this direction, the present study adopted a contactless RGB video-based sensing protocol aligned with SPPB tasks to improve usability and scalability in older adult screening.

The assessment protocol lasted approximately 90 s and consisted of the following three subtests:

- Balance subtest: Participants completed standing balance tasks, including side-by-side stance, semi-tandem stance, and tandem stance according to the SPPB testing sequence.
- Chair-stand subtest: Participants performed a five-time sit-to-stand task. The five-times sit-to-stand test has been reported as a predictor of recurrent falls in healthy community-living adults aged 65 years and older [19].
- Gait subtest: Participants completed a 3 m usual-pace walking test. Gait speed was calculated as:

(1) $$v_{gait}=\frac{3}{t_{walk}}(m/s),$$

where $v_{gait}$ (m/s) denotes gait speed and $t_{walk}$ (s) is the time required to traverse the 3 m distance, following established SPPB procedures.

The SPPB-aligned assessment tasks served as the clinical functional reference framework for constructing the operational functional risk index. Because prospective fall-event outcomes were not available in this cross-sectional dataset, the supervised learning target was not constructed from observed future falls. Instead, the labels were generated from an SPPB-aligned functional risk index described in the following section. Accordingly, the proposed model should be interpreted as performing SPPB-aligned functional fall-risk stratification rather than validated prediction of prospective fall occurrence.

### 2.3. Construction of the SPPB-Aligned Functional Risk Index and Five-Level Labels

Because prospective fall-event outcomes were not available in this cross-sectional dataset, the supervised labels were not constructed from observed future falls. Instead, a pre-specified SPPB-aligned functional risk index was used to generate the five-level classification labels. This index was established before model development as an operational scoring scheme to summarize functional mobility impairment and dynamic postural-control burden during SPPB-aligned assessment tasks. Therefore, the index should be interpreted as a functional stratification score rather than as a calibrated probability of prospective fall occurrence.

The functional risk index ranged from 0 to 100 and consisted of five components: gait speed, five-times sit-to-stand performance, balance-task completion, left lateral trunk deviation angle, and right lateral trunk deviation angle. The component structure and maximum contribution of each indicator are summarized in Table 1. The three SPPB-aligned task-performance components accounted for 60 points of the index. Gait speed contributed up to 30 points because walking performance is a central indicator of functional mobility. Five-times sit-to-stand performance and balance-task performance contributed up to 15 points each, reflecting lower-limb transitional function and static postural control, respectively.

The remaining 40 points were derived from vision-based skeletal motion features. Specifically, the left lateral trunk deviation angle and right lateral trunk deviation angle contributed up to 20 points each. These features quantified leftward and rightward trunk deviation relative to the core midline during walking and were included to capture mediolateral trunk-control characteristics that are not directly quantified by conventional SPPB scoring. These angular deviation features were interpreted as relative two-dimensional mediolateral trunk-control indicators derived from standardized frontal-view skeletal trajectories rather than as absolute three-dimensional trunk kinematics.

The component weights were pre-specified before model training and were not optimized using validation or test-set classification results. After the five component scores were summed, the total functional risk index was converted into five ordinal supervised-learning labels. The mapping between the functional risk index and the five label levels is shown in Table 2. Index values of ≤5%, >5% to ≤17.5%, >17.5% to ≤30%, >30% to ≤50%, and >50% were assigned to Levels 1–5, respectively. These five ordinal labels were used as the supervised learning targets in this study. The detailed task-level conversion rules were implemented according to the pre-specified scoring protocol used to construct the functional risk index.

### 2.4. RGB Video-Based Sensing Setup and Two-Dimensional Motion Representation

Motion data were acquired using a single monocular RGB camera placed approximately 3 m in front of the participant at a fixed height of 95 cm. The camera was positioned to capture a standardized frontal full-body view throughout all assessment tasks. Video was recorded at a resolution of 1920 × 1080 pixels and a frame rate of 30 frames/s. This sensing configuration was designed to emulate community-based functional screening conditions while reducing participant burden compared with wearable sensing systems. Previous work has identified multiple sensor-based parameters associated with fall risk in community-dwelling older adults, supporting the use of objective sensing-derived movement features for fall-risk interpretation [20]. Building on this sensing-oriented perspective, the present study used a contactless monocular RGB setup to extract skeletal motion features during standardized SPPB-aligned functional mobility tasks.

All assessment tasks, including balance, five-times sit-to-stand, and gait testing, were recorded from the frontal-view perspective. Therefore, the extracted skeletal trajectories represented two-dimensional image-plane motion patterns obtained under a standardized camera configuration.

The proposed framework did not attempt to reconstruct absolute three-dimensional joint trajectories from monocular RGB video. Instead, it used frontal-view two-dimensional skeletal trajectories as normalized image-plane kinematic representations. Pelvis-centered translation and height-based scaling were subsequently applied to reduce variability caused by camera distance, participant body size, and initial standing location. Accordingly, the extracted skeletal features should be interpreted as relative two-dimensional motion descriptors rather than absolute three-dimensional biomechanical measurements.

The left and right lateral trunk deviation angles used in the functional risk index were also derived from the frontal-view skeletal representation. These angles described trunk lean relative to the estimated core midline during walking and were intended to characterize mediolateral trunk-control behavior. Because they were computed from two-dimensional skeletal trajectories, they should be interpreted as standardized frontal-view mediolateral deviation indicators rather than full three-dimensional trunk kinematic variables.

**Figure 1:**
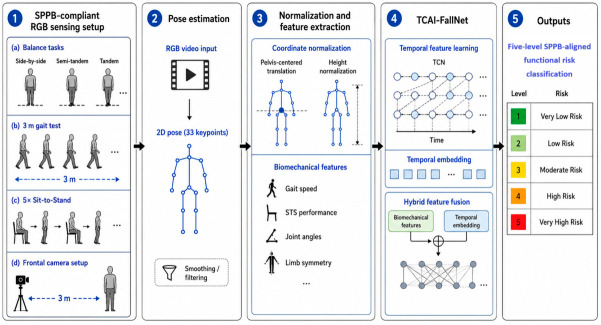
Proposed contactless SPPB-aligned functional risk assessment framework and SPPB-compliant RGB sensing setup. (1) SPPB-compliant RGB sensing setup for balance, 3 m gait, and five-times sit-to-stand tasks under a standardized frontal camera configuration; (2) pose estimation from RGB video to obtain 33 two-dimensional skeletal keypoints with smoothing and filtering; (3) coordinate normalization and biomechanical feature extraction, including pelvis-centered translation, height normalization, gait speed, sit-to-stand performance, joint-angle features, and limb-symmetry descriptors; (4) TCAI-FallNet-based temporal modeling and hybrid feature fusion, combining TCN-derived temporal embeddings with handcrafted biomechanical descriptors; and (5) five-level SPPB-aligned functional risk classification output.

The overall RGB video-based sensing setup and processing workflow are illustrated in Figure 1. The proposed framework consists of five sequential processes. First, SPPB-compliant RGB video acquisition was performed during balance tasks, the 3 m gait assessment, and the five-times sit-to-stand task under a standardized frontal camera setup. Second, RGB video frames were processed using BlazePose (MediaPipe, Google LLC, Mountain View, CA, USA) to extract 33 two-dimensional skeletal keypoints, followed by smoothing and filtering to improve trajectory stability. Third, skeletal coordinates were normalized using pelvis-centered translation and height-based scaling, and biomechanical descriptors, including gait speed, sit-to-stand performance, joint-angle features, and limb-symmetry-related indicators, were extracted. Fourth, TCAI-FallNet was used to learn temporal skeletal representations through a Temporal Convolutional Network and to fuse TCN-derived embeddings with handcrafted biomechanical descriptors. Fifth, the fused representation was classified into five SPPB-aligned functional risk levels.

### 2.5. Skeletal Keypoint Extraction and Quality Control

For each video frame, two-dimensional skeletal keypoints were extracted using BlazePose. BlazePose was selected as the front-end pose estimation model because it provides a practical balance among computational efficiency, real-time performance, and anatomical keypoint coverage. Although high-resolution pose estimation models can provide strong localization performance, their computational requirements may limit real-time deployment in community-based or edge-oriented screening environments. In contrast, lightweight pose estimation pipelines are more suitable for scalable functional mobility assessment.

Prior AI-based fall evaluation research using gesture detection of gait and balance has shown the feasibility of applying computer vision and artificial intelligence to community-dwelling older adult assessment [21]. In addition, validation studies using OpenPose and RGB webcams have supported the feasibility of markerless gait analysis under video-based sensing conditions [22]. Therefore, the present framework adopted markerless skeletal tracking as the basis for dynamic postural feature extraction.

For each video frame *t*, BlazePose outputs a set of 33 anatomical keypoints, and the skeletal representation matrix $K_{t}$ was defined as

(2) $$K_{t}={\left[p_{1},p_{2},\ldots ,p_{33}\right]}^{T}\in R^{33\times 2},$$

where each row $p_{i}=(x_{i},y_{i})$ corresponds to the two-dimensional image-plane coordinates of the i-th anatomical joint. Coordinates are expressed in pixel units under the camera image reference frame, with the origin located at the top-left corner of the image, the *x*-axis oriented horizontally to the right, and the *y*-axis oriented vertically downward.

To improve the reliability of skeletal trajectory extraction, a sequence-level quality-control procedure was applied before model training and evaluation. Videos were reviewed for task completeness, frontal-view visibility, and major occlusion before analysis. Sequences that could not support reliable skeletal trajectory extraction were excluded from model development and evaluation. For retained videos, short and isolated keypoint discontinuities were corrected using temporal interpolation, and frame-wise skeletal jitter was reduced using temporal smoothing before feature extraction. Keypoint detection success was defined as the proportion of frames in which all required trunk and lower-limb landmarks exceeded the predefined confidence threshold of 0.5. Frame-wise jitter was quantified as the mean frame-to-frame displacement of normalized skeletal landmarks after removing gross task-related motion trends. This quality-control procedure was intended to reduce noise in the two-dimensional skeletal trajectories rather than to infer unobserved three-dimensional joint motion.

### 2.6. Skeletal Coordinate Normalization

To reduce variability caused by camera position, participant body size, and initial standing location, skeletal coordinates were normalized using pelvis-centered translation and height-based scaling. This procedure was applied to the two-dimensional frontal-view skeletal trajectories extracted from the monocular RGB videos.

First, let $k_{t,j}$ denote the coordinate vector of joint *j* at frame *t*, and let $k_{t,hip}$ represent the midpoint between the left and right hip joints. Pelvis-centered coordinates were computed as

(3) $$\tilde{k_{t,j}}=k_{t,j}-k_{t,hip},$$

where $\tilde{k_{t,j}}\;$ is the pelvis-centered coordinate vector of joint *j* at frame *t*, $k_{t,j}\;$ is the original global coordinate vector, and $k_{t,hip}$ denotes the coordinate vector of the root (midpoint of hips).

**Figure 2:**
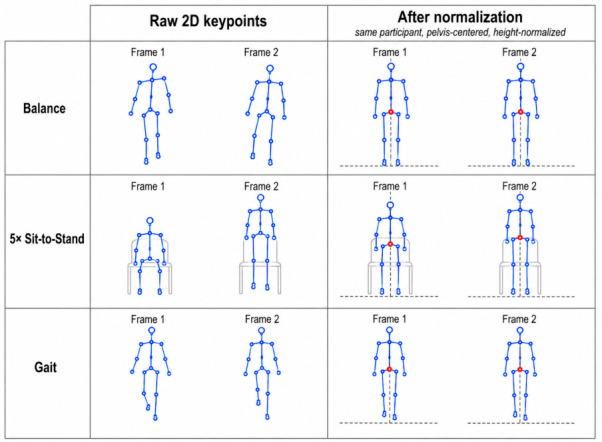
Comparison of unnormalized and normalized skeletal trajectories. Blue points and lines indicate two-dimensional skeletal keypoints and body-segment connections; red points indicate pelvis-centered reference landmarks after normalization; gray dashed lines indicate visual reference lines for alignment.

Second, height-based scaling was applied to compensate for inter-subject anthropometric variability by normalizing skeletal coordinates using a participant-specific reference body height. Let hi denote the participant-specific reference body height estimated from stable upright frames, defined as the median vertical distance between the neck and mid-ankle landmarks during standing frames. This subject-level reference height was used for all frames of the participant to reduce body-size variability while preserving within-subject temporal changes such as sit-to-stand transitions. The scale-normalized coordinates are defined as

(4) $$\hat{k_{t,j}}=\frac{\tilde{k_{t,j}}}{h_{i}},$$

where $\hat{k_{t,j}}$ represents the final scale-normalized coordinate vector of joint *j* at frame *t*, $\tilde{k_{t,j}}$ is the pelvis-centered coordinate vector derived from Equation (3), and $h_{i}$ is the participant-specific reference body height. This normalization procedure reduced global translation and body-size-related variability while preserving relative skeletal configurations and temporal motion patterns. The comparison between unnormalized and normalized skeletal trajectories is illustrated in Figure 2.

### 2.7. Biomechanical Feature Engineering

In addition to learned temporal embeddings, interpretable biomechanical descriptors were extracted to characterize postural stability, lower-limb control, and gait regularity. These descriptors were selected because they correspond to movement patterns that are clinically relevant to functional mobility assessment and can be derived from standardized frontal-view two-dimensional skeletal trajectories.

#### 2.7.1. Center-of-Mass Mediolateral (CoM) Sway

Mediolateral center-of-mass sway was estimated using trunk-related skeletal keypoints:

(5) $${CoM}_{ML}(t)=\frac{1}{N}\sum_{j\in T}{\hat{x}}_{t,j},$$

where *T* denotes the set of trunk joints, *N* is the number of joints in *T*, and ${\hat{x}}_{t,j}\;$ represents the normalized horizontal coordinate of the joint j. The sway path length was computed as

(6) $$L_{COM}=\frac{1}{T-1}\sum_{t=2}^{T}\left|{\mathrm{CoM}}_{ML}\left(t\right)-{\mathrm{CoM}}_{ML}\left(t-1\right)\right|.$$

The value was normalized by sequence length to allow comparison across participants with different task durations. Greater mediolateral sway was interpreted as greater instability in lateral postural control during the assessment sequence.

#### 2.7.2. Sit-to-Stand Temporal Efficiency

For the chair-stand subtest, sit-to-stand temporal efficiency was quantified using the duration of movement execution. The sit-to-stand duration was calculated as:

(7) $$T_{\mathrm{STS}}=t_{stand}-t_{sit},$$

where $t_{sit}$ and $t_{stand}$ represent the detected onset and completion timestamps of the sit-to-stand movement. The STS time ratio was calculated as the participant’s five-times sit-to-stand duration divided by the predefined reference duration used in the SPPB-aligned scoring rule. Longer sit-to-stand duration was interpreted as reduced lower-limb transitional function.

#### 2.7.3. Hip–Knee Angular Asymmetry

Hip–knee angular asymmetry was used to characterize bilateral lower-limb coordination. Joint angles were estimated using normalized skeletal coordinates. The hip–knee angular asymmetry was defined as

(8) $$A_{\mathrm{HK}}=\frac{1}{T}\sum_{t=1}^{T}\left|\theta_{t}^{\left(L\right)}-\theta_{t}^{\left(R\right)}\right|,$$

where $\theta_{t}^{\left(L\right)}$ and $\theta_{t}^{\left(R\right)}$ represent left and right joint angles at frame *t*, respectively, and $A_{\mathrm{HK}}$ represents the mean bilateral angular asymmetry across the assessment sequence. Increased asymmetry may reflect impaired neuromuscular coordination or compensatory movement strategies.

#### 2.7.4. Stride-Time Variability

Stride-time variability was extracted from the gait sequence to characterize temporal irregularity during walking. Let *ST_i_* denote the estimated stride time of the *i*-th gait cycle. Stride-time variability was calculated as:

(9) $$STV=\frac{\sigma (ST)}{\mu (ST)},$$

where σ(*ST*) and μ(*ST*) denote the standard deviation and mean of stride time, respectively. Higher stride-time variability indicates less stable gait rhythm and was used as an indicator of gait irregularity.

The engineered biomechanical descriptors were used together with learned temporal embeddings in the hybrid classification framework. These descriptors were not interpreted as absolute three-dimensional biomechanical measurements; rather, they represented relative two-dimensional movement indicators extracted under a standardized frontal-view sensing configuration.

### 2.8. Temporal Convolutional Network Architecture for Skeletal Sequence Modeling

The proposed TCAI-FallNet framework used a Temporal Convolutional Network (TCN) to model sequential skeletal motion patterns. The model input consisted of normalized skeletal coordinates across time. The network processes an input skeletal sequence spanning *T* frames within a single assessment window. Specifically, the input at each time step is a 66-dimensional feature vector constructed by flattening the scale-normalized two-dimensional coordinates of all 33 body joints derived in the previous section.

In this study, the temporal input sequence was derived from the complete SPPB-aligned assessment protocol, including static balance tests, five-times sit-to-stand, and the 3 m gait speed task. Task-wise segmentation was avoided in the primary model to preserve cross-task temporal continuity and prevent error propagation from task-boundary detection. This design enabled the model to learn unified temporal representations across balance control, transitional movement, and walking patterns.

**Figure 3:**
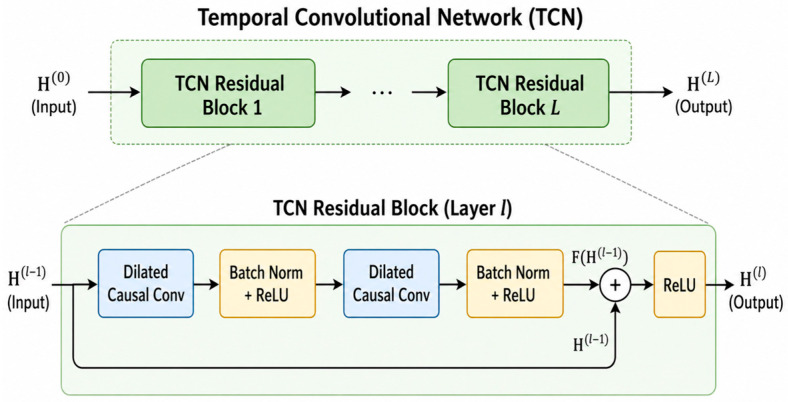
Architecture of the proposed Temporal Convolutional Network for skeletal motion sequence modeling.

Each TCN layer performs a dilated one-dimensional convolution along the temporal dimension, defined as

(10) $$H^{\left(l\right)}=ReLU\left(W^{\left(l\right)}*H^{\left(l-1\right)}\right),$$

where $H^{\left(l\right)}$ denotes the output feature map of the *l*-th layer, $W^{\left(l\right)}$ represents the learnable convolutional kernel weights. The operator “∗” denotes a one-dimensional dilated convolution operator, whose dilation configuration increases exponentially with layer depth. This design enables rapid expansion of the temporal receptive field while preserving temporal resolution. The activation function is implemented using the Rectified Linear Unit (ReLU). Equation (10) provides a simplified representation of the temporal convolution operation; in implementation, each residual block consisted of two dilated causal convolution layers followed by batch normalization and ReLU activation, with a residual skip connection, as illustrated in Figure 3. This design enabled rapid expansion of the temporal receptive field while preserving temporal resolution.

Accordingly, the effective temporal receptive field *R* of the TCN is given by

(11) $$R=1+(k-1)\sum_{l=1}^{L}2^{l-1},$$

where *k* is the kernel size and *L* is the number of convolutional layers. In this implementation, the kernel size and the number of convolutional layers were set to k = 3 and L = 8, respectively. This configuration yielded an effective receptive field of 511 frames. Rather than assuming full-sequence coverage, the receptive field was designed to capture extended postural and gait-related temporal dependencies while maintaining a compact computational structure. This allowed the proposed architecture to model short-term motion fluctuations, such as mediolateral sway during balance tests, as well as longer temporal patterns, such as gait rhythm and sit-to-stand transitions, within a unified temporal representation. Compared with recurrent or transformer-based architectures, the proposed TCN design provides competitive temporal modeling capacity with lower computational cost and inference latency, making it suitable for real-time functional risk stratification systems. The architecture of the proposed TCN-based temporal modeling framework is illustrated in Figure 3.

### 2.9. Hybrid Feature Fusion and Classification

To combine learned temporal representations with interpretable biomechanical information, the final TCN embedding $z_{TCN}$ was concatenated with the biomechanical feature vector $z_{bio}$ as

(12) $$z=\left[z_{bio}|z_{TCN}\right].$$

The fused representation (z) was then passed to a fully connected classification layer followed by a softmax function to estimate the probability distribution across the five SPPB-aligned functional risk levels:

(13) $$\hat{y_{i}}=\frac{\exp\left(w_{i}^{⊤}z\right)}{\sum_{j=1}^{C}\exp\left(w_{j}^{⊤}z\right)},$$

where $\hat{y_{i}}\;$ represents the predicted probability that the input belongs to the *i*-th SPPB-aligned functional risk category, and exp(·) is the exponential operation. The term $w_{i}$ denotes the learnable weight vector corresponding specifically to class *i*, and z is the fused feature vector derived from Equation (12). The denominator serves as a normalization factor, summing the exponentials over all *C* = 5 classes (indexed by *j*) to ensure that the resulting probabilities across all categories sum to one. The final predicted label was assigned according to the class with the highest softmax probability.

This hybrid modeling strategy was designed to combine the discriminative capacity of learned temporal representations with the interpretability of clinically meaningful biomechanical descriptors. To evaluate whether the performance gain was attributable to the proposed fusion design rather than to an unfair comparison against sequence-only baselines, additional ablation analyses were conducted using biomechanical-only, TCN-only, and fused representations.

### 2.10. Data Partitioning and Participant-Level Model Validation

To reduce the risk of data leakage, data partitioning was performed at the participant level. All video sequences, skeletal trajectories, biomechanical descriptors, and derived labels belonging to the same participant were assigned exclusively to one subset and were not shared across the training, validation, and test sets. This participant-independent partitioning ensured that model performance reflected generalization to unseen individuals rather than repeated observations or multiple task segments from the same participant.

The dataset of 688 participants was divided into training, validation, and test sets using a stratified participant-level split with a ratio of approximately 60%/20%/20%. The training set included 412 participants and was used for model parameter learning. The validation set included 137 participants and was used for hyperparameter tuning and early stopping. The test set included 139 participants and was kept untouched until final model evaluation. No participant appeared in more than one subset.

The class distribution of the full cohort and each data subset is summarized in Table 3. This table was added to improve transparency regarding class imbalance and to support the interpretation of macro-averaged evaluation metrics. The class distribution across the five SPPB-aligned functional risk levels was relatively balanced. In the full cohort, Level 1 included 128 participants (18.6%), Level 2 included 146 participants (21.2%), Level 3 included 122 participants (17.7%), Level 4 included 151 participants (22.0%), and Level 5 included 141 participants (20.5%). The percentages shown in the training, validation, and test columns represent the proportion of participants within each functional risk level assigned to each subset.

The split was stratified by functional risk level to preserve comparable class proportions across subsets. Hyperparameters were selected based only on validation-set performance, and the test set was not used during model selection, threshold selection, class-weight calculation, or early stopping.

### 2.11. Baseline Models and Fair Comparison Settings

To evaluate the effectiveness of the proposed TCAI-FallNet architecture, the model was compared with representative classic and lightweight temporal baseline architectures. The classic baseline models included CNN, CNN-LSTM, ConvLSTM, and Transformer models. These models were selected to represent spatial–temporal convolutional modeling, recurrent temporal modeling, convolutional recurrent modeling, and attention-based temporal modeling, respectively.

To further compare the proposed model with lightweight temporal architectures, additional temporal convolutional variants were included, namely TCN-only, depthwise-separable TCN (DS-TCN), and multi-scale TCN-lite (MS-TCN-lite). The TCN-only model was used to evaluate the contribution of learned temporal skeletal embeddings without handcrafted biomechanical descriptors. DS-TCN was included as a parameter-efficient temporal convolutional baseline using depthwise-separable convolutional operations. MS-TCN-lite was included to evaluate whether multi-scale temporal convolutional modeling could improve sequence representation while maintaining a compact model structure. In DS-TCN, standard temporal convolutional layers were replaced by depthwise-separable temporal convolutions. In MS-TCN-lite, multiple lightweight temporal convolution branches with different dilation settings were fused before classification.

To ensure fair comparison, all baseline, lightweight temporal, and ablation models were trained and evaluated using the same participant-level data partitioning, supervised labels, preprocessing protocol, optimizer, learning rate, batch size, early-stopping criterion, and class-weighting strategy unless otherwise specified. All sequence-based baseline models received the same normalized skeletal coordinate sequences as input. The proposed TCAI-FallNet additionally incorporated handcrafted biomechanical descriptors through late feature fusion.

To avoid overstating the contribution of the hybrid design, additional ablation variants were included: a biomechanical-only classifier, a TCN-only model using skeletal sequences without handcrafted descriptors, and the full fused TCAI-FallNet model. The biomechanical-only classifier was used to assess the discriminative value of handcrafted descriptors alone, whereas the TCN-only model was used to assess the contribution of learned temporal skeletal embeddings alone. The full fused TCAI-FallNet model was then evaluated to determine whether combining handcrafted biomechanical descriptors with TCN-derived temporal embeddings improved five-level SPPB-aligned functional risk classification.

For all baseline and ablation models, model training was conducted using the same training set, hyperparameter selection was performed using the validation set, and final performance was reported on the held-out participant-independent test set. This design ensured that the comparison between TCAI-FallNet and baseline models was based on the same participant-level evaluation protocol. The input features and comparison settings for all models are summarized in Table 4.

### 2.12. Model Training and Optimization

The model was trained using a weighted categorical cross-entropy loss to address class imbalance across the five SPPB-aligned functional risk levels:

(14) $$L=-\sum_{c=1}^{5}w_{c}\;y_{c}\;\log\left(\hat{y_{c}}\right),$$

where $y_{c}$ denotes the ground-truth label indicator for class *c*, $\hat{y_{c}}$ denotes the predicted probability for class *c*, and $w_{c}$ is the class weight inversely proportional to class frequency in the training set. The Adam optimizer was used with an initial learning rate of 0.001. The batch size was set to 32, and the maximum number of training epochs was set to 150. Early stopping was applied based on validation macro-AUC with a patience of 20 epochs to reduce overfitting. The best model checkpoint was selected according to validation performance and was subsequently evaluated once on the held-out participant-independent test set.

Class weights were calculated using the class distribution of the training set only. Validation and test data were not used to estimate class weights or tune model parameters. This procedure was adopted to preserve the independence of model evaluation.

### 2.13. Evaluation Metrics and Statistical Analysis

To comprehensively evaluate the proposed five-level SPPB-aligned functional risk classification under potential class imbalance, multiple performance metrics were employed, including macro-averaged area under the receiver operating characteristic curve (macro-AUC), precision, and recall. The macro-AUC was adopted to provide an unbiased assessment across all risk levels by equally weighting each class-specific ROC curve, defined as

(15) $${\mathrm{AUC}}_{macro}=\frac{1}{C}\sum_{i=1}^{C}{\mathrm{AUC}}_{i},$$

where *C* = 5 denotes the functional risk levels and ${\mathrm{AUC}}_{i}$ represents the area under the ROC curve for the *i*-th class. Macro-averaging was selected to mitigate dominance effects from majority classes and to ensure fair evaluation across all risk levels. Class-wise precision and recall were computed as

(16) $${\mathrm{Precision}}_{c}=\frac{TP_{c}}{TP_{c}+FP_{c}}.$$

(17) $${\mathrm{Recall}}_{c}=\frac{TP_{c}}{TP_{c}+FN_{c}},$$

where $TP_{c}$, $FP_{c},$ and $FN_{c}\;$ denote the number of true positives, false positives, and false negatives for class *c*, respectively. Macro-averaged precision and recall were subsequently obtained by averaging the per-class values, providing balanced performance estimates under imbalanced class distributions. The class-wise F1-score was calculated as the harmonic mean of precision and recall, and macro-F1 was obtained by averaging the F1-scores across the five functional risk levels.

For biomechanical feature comparisons across the five functional risk levels, continuous variables were summarized as mean ± standard deviation. Because the distributions of biomechanical descriptors were not assumed to be normally distributed across all five levels, between-level differences were evaluated using the Kruskal–Wallis test. A *p*-value less than 0.05 was considered statistically significant. When applicable, post hoc pairwise comparisons were conducted with multiple-comparison adjustment.

For model comparison, all performance metrics were calculated on the held-out participant-independent test set. The ablation analysis was used to examine the incremental contribution of biomechanical descriptors, learned temporal embeddings, skeletal normalization, and class weighting. Because the primary evaluation used a single held-out participant-level test set, inferential statistical comparison across repeated folds was not performed; this limitation was acknowledged in the Section 4.

### 2.14. SHAP-Based Explainability Analysis

To assess model interpretability, SHapley Additive exPlanations (SHAP) were applied to the trained classifier. SHAP is a post hoc feature attribution method based on cooperative game theory and has been widely used to interpret complex machine-learning models [23]. In this study, SHAP analysis was used to quantify the relative contribution of biomechanical descriptors and learned temporal representations to the predicted SPPB-aligned functional risk levels.

SHAP values were computed using the final trained fused classifier. Background reference samples were drawn from the training set to represent the empirical distribution of the input features, while attribution values were evaluated on the held-out participant-independent test set. Because the TCN-derived embeddings are latent temporal representations and are not directly equivalent to clinical variables, clinical interpretation focused primarily on the handcrafted biomechanical descriptors, including mediolateral center-of-mass sway, sit-to-stand time ratio, hip–knee angular asymmetry, and stride-time variability.

Mean absolute SHAP values were used to rank global feature importance. In addition, SHAP dependence analyses were performed for the top-ranked biomechanical descriptors to examine whether increasing feature values were associated with shifts toward higher functional risk levels. These analyses were intended to support post hoc interpretability rather than to establish causal relationships or clinically validated decision thresholds.

Because the final classifier received a fused tabular representation consisting of handcrafted biomechanical descriptors and TCN-derived embeddings, SHAP values were estimated using a model-agnostic Kernel SHAP procedure with training-set background samples.

### 2.15. Implementation and Inference-Latency Assessment

The proposed framework was implemented using PyTorch 2.1 (Meta AI, Menlo Park, CA, USA) and evaluated on a workstation equipped with an Intel i5-9500E CPU (Intel Corporation, Santa Clara, CA, USA) and an NVIDIA A4000 GPU (NVIDIA Corporation, Santa Clara, CA, USA). All inference experiments were conducted under identical runtime conditions to ensure consistent performance measurement. The system was designed following lightweight convolutional neural network principles commonly adopted in embedded and edge-oriented AI applications. In particular, the architectural design draws inspiration from inverted residual blocks and linear bottlenecks, which have been shown to reduce computational cost and model size while preserving representational capacity [24].

The final trained TCAI-FallNet model contained approximately 1.28 million parameters and had a serialized model size of 2.8 MB. The reported inference latency of approximately 19–20 ms per input sequence refers specifically to TCN model inference after pose estimation, coordinate normalization, and feature extraction. It does not include video acquisition, BlazePose front-end processing, file input/output, user-interface rendering, report generation, or network transmission.

Therefore, the computational results should be interpreted as evidence of lightweight TCN inference and future edge-oriented implementation potential rather than full end-to-end edge–device validation. End-to-end latency on representative embedded devices, smartphones, or low-power edge hardware remains to be evaluated in future work.

## 3. Results

### 3.1. Dataset Summary and Preprocessing Outcomes

After quality screening, 688 participants were included for model development and evaluation. The mean participant age was 74.3 ± 6.2 years, with an age range of 65–92 years. The average sequence lengths for the standing balance, sit-to-stand, and gait tasks were 309 ± 12, 182 ± 25, and 210 ± 33 frames, respectively. Although the full assessment session lasted approximately 90 s, only task-active video segments corresponding to balance, sit-to-stand, and gait execution were retained for skeletal sequence modeling. The dataset was divided using a stratified participant-level 60%/20%/20% split. The training set included 412 participants, the validation set included 137 participants, and the held-out test set included 139 participants. No participant appeared in more than one subset. The class distribution across the five SPPB-aligned functional risk levels is reported in Table 3. The demographic and sequence-level characteristics of the dataset are summarized in Table 5.

Across all retained videos, the skeletal keypoint detection success rate exceeded 98.4%. Temporal smoothing reduced frame-wise joint jitter by 21.3% relative to unsmoothed pose trajectories, resulting in more stable skeletal representations for downstream feature extraction and temporal modeling.

### 3.2. Effects of Skeletal Coordinate Normalization

Root-centered translation and height-based normalization were applied to reduce inter-subject variability caused by differences in body size, camera distance, and initial standing position. The quantitative effects of normalization on trajectory consistency are reported below.

After normalization, the mediolateral displacement variance of the estimated center-of-mass trajectory decreased by 34.7% across participants. In addition, the average inter-subject correlation of joint trajectories increased by 29.1%. These results indicate that the normalization procedure improved cross-subject alignment and provided a more stable input representation for biomechanical feature extraction and temporal convolutional modeling.

### 3.3. Biomechanical Feature Differences Across Functional Risk Levels

The proposed biomechanical descriptors demonstrated consistent differences across the five SPPB-aligned functional risk levels. As shown in Table 6, mediolateral center-of-mass sway increased monotonically from Level 1 to Level 5, indicating progressive deterioration of lateral postural stability with increasing functional risk. The sit-to-stand time ratio also increased across risk levels, reflecting reduced lower-limb power and impaired transitional movement control in higher-risk participants.

Hip–knee angular asymmetry and stride-time variability also showed increasing trends across the five SPPB-aligned functional risk levels. The largest differences were observed between the lower-risk groups and the higher-risk groups, suggesting that bilateral lower-limb coordination and gait rhythm irregularity were important indicators of functional decline. These findings support the physiological relevance of the selected biomechanical descriptors and justify their integration into the hybrid classification model.

These results support the physiological relevance of the selected descriptors and justify their integration into the hybrid classification model. In particular, the monotonic trends in mediolateral sway and stride-time variability suggest that dynamic postural control and gait rhythm irregularity were associated with higher SPPB-aligned functional risk.

### 3.4. Classification Performance

The proposed TCAI-FallNet model was compared with representative classic and lightweight temporal baseline architectures. The classic baseline models included CNN, CNN-LSTM, ConvLSTM, and Transformer architectures. These models were selected to represent spatial–temporal convolutional modeling, recurrent temporal modeling, convolutional recurrent modeling, and attention-based temporal modeling, respectively. To further contextualize the lightweight temporal modeling capacity of the proposed architecture, additional temporal baselines were included, namely TCN-only, depthwise-separable TCN (DS-TCN), and multi-scale TCN-lite (MS-TCN-lite). The TCN-only model evaluated the contribution of learned temporal skeletal embeddings without handcrafted biomechanical descriptors, whereas DS-TCN and MS-TCN-lite were included as lightweight temporal convolutional variants. All models were trained and evaluated using the same participant-level data split, preprocessing protocol, class-weighting strategy, optimizer setting, learning rate, batch size, and early-stopping criterion. Sequence-based baseline models received the same normalized skeletal coordinate sequence as input. The proposed TCAI-FallNet further incorporated handcrafted biomechanical descriptors through late feature fusion to evaluate whether the integration of interpretable biomechanical features and learned temporal embeddings improved five-level SPPB-aligned functional risk classification.

As summarized in Table 7, the proposed TCAI-FallNet achieved the strongest overall performance across macro-averaged metrics, with a macro-F1 score of 0.82, macro-precision of 0.84, macro-recall of 0.81, macro-AUC of 0.91, and overall accuracy of 81.3% on the held-out participant-independent test set. Compared with the CNN baseline, TCAI-FallNet improved macro-F1 from 0.71 to 0.82 and macro-AUC from 0.83 to 0.91. Compared with recurrent and attention-based baselines, including CNN-LSTM, ConvLSTM, and Transformer, the proposed model achieved higher balanced performance while maintaining a compact parameter count.

Among the lightweight temporal baselines, the TCN-only model achieved a macro-F1 of 0.78 and a macro-AUC of 0.88, indicating that temporal convolutional modeling captured meaningful sequential skeletal patterns. DS-TCN achieved a smaller parameter count but lower classification performance, suggesting that extreme parameter reduction may reduce temporal representation capacity. MS-TCN-lite achieved stronger performance than TCN-only and DS-TCN, but still remained below the proposed fused model. These results suggest that the proposed TCAI-FallNet achieved a favorable balance between model compactness, temporal modeling capacity, and classification performance by combining TCN-derived temporal embeddings with handcrafted biomechanical descriptors.

The performance gain of the proposed model indicates that combining interpretable biomechanical descriptors with TCN-derived temporal embeddings improved five-level SPPB-aligned functional risk classification. The improvement in macro-averaged metrics suggests that the model did not rely solely on majority-class patterns.

### 3.5. Ablation Study

To examine the contribution of each major design component, ablation analyses were conducted using biomechanical-only features, TCN-only embeddings, and the full fused TCAI-FallNet representation. Additional ablations were performed to evaluate the effects of skeletal normalization, temporal smoothing, class weighting, and full-sequence temporal modeling.

As shown in Table 8, the fused TCAI-FallNet model achieved higher macro-F1 and macro-AUC than either the biomechanical-only or TCN-only variants. The biomechanical-only classifier achieved a macro-AUC of 0.80, indicating that the handcrafted descriptors carried clinically meaningful information but were insufficient alone. The TCN-only model achieved a macro-AUC of 0.88, suggesting that learned temporal embeddings captured important sequential skeletal patterns. The full fused model further improved macro-AUC to 0.91, supporting the complementary contribution of handcrafted biomechanical descriptors and learned temporal representations.

Removing skeletal normalization, temporal smoothing, or class weighting reduced model performance. The task-wise segmented input variant also performed slightly worse than the full-sequence input, suggesting that preserving cross-task temporal continuity contributed to model performance.

### 3.6. Sensitivity Analysis of the Functional Risk Index Weighting Scheme

Because the five-level labels were derived from a pre-specified SPPB-aligned functional risk index, a sensitivity analysis was conducted to evaluate whether the label stratification and model performance were overly dependent on the selected component-weighting scheme. The proposed operational weighting scheme was compared with two alternative strategies: an equal-weight scheme and an SPPB-dominant scheme.

As summarized in Table 9, the alternative weighting schemes showed high agreement with the original label structure. The equal-weight scheme achieved an agreement rate of 88.2% and a weighted kappa of 0.84 relative to the proposed operational scheme. The SPPB-dominant scheme achieved an agreement rate of 86.9% and a weighted kappa of 0.82. Model performance remained broadly consistent across alternative weighting strategies, suggesting that the main conclusions were not driven solely by the original 60/40 weighting structure.

These findings indicate that the functional risk stratification was reasonably stable under alternative weighting assumptions. Nevertheless, the proposed index should still be interpreted as an operational functional stratification scheme rather than as a clinically calibrated fall-event probability model.

### 3.7. ROC Analysis

**Figure 4:**
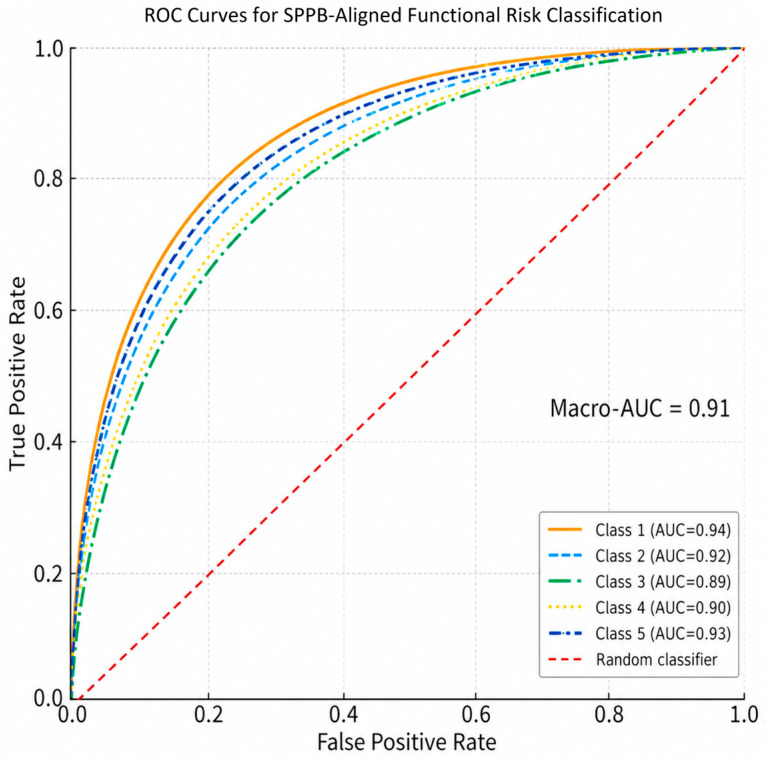
Receiver operating characteristic curves for five-level SPPB-aligned functional risk classification.

Receiver operating characteristic analysis was conducted to evaluate the discriminative performance of the proposed TCAI-FallNet model across the five SPPB-aligned functional risk levels. The one-versus-rest ROC curves are shown in Figure 4. The proposed model achieved class-wise AUC values of 0.94, 0.92, 0.89, 0.90, and 0.93 for Levels 1–5, respectively. The resulting macro-averaged AUC was 0.91.

The ROC curves demonstrated balanced discrimination across both low-risk and high-risk categories. Importantly, the model maintained strong separability for Level 1 and Level 5, indicating reliable identification of participants at the two extremes of the functional risk spectrum.

### 3.8. Confusion Matrix Analysis

**Figure 5:**
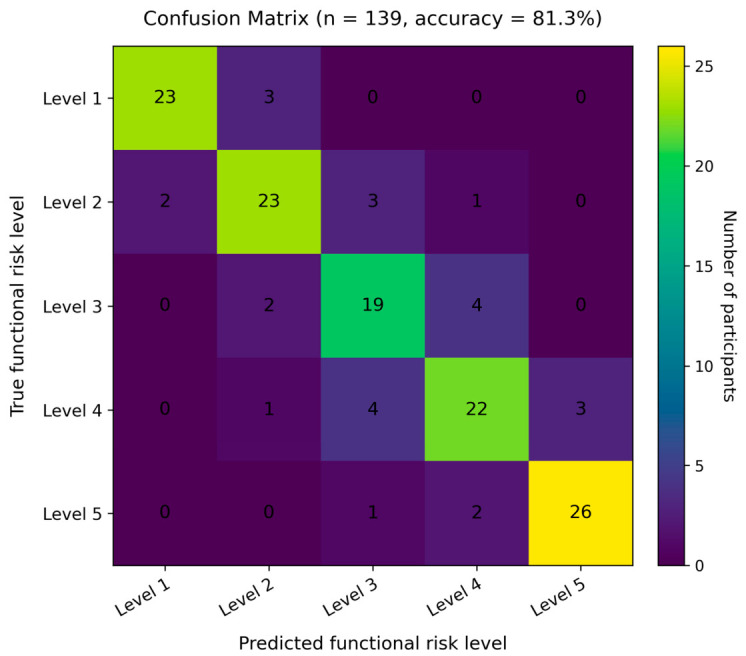
Confusion matrix of the proposed TCAI-FallNet model for five-level SPPB-aligned functional risk classification.

The confusion matrix analysis further examined the distribution of predicted and true SPPB-aligned functional risk levels in the held-out participant-independent test set. As shown in Figure 5, the proposed model correctly classified 113 of 139 test participants, corresponding to an overall accuracy of 81.3%. The model achieved strong classification performance at the two ends of the functional risk spectrum, with correct classification rates of 88.5% for Level 1 and 89.7% for Level 5.

Most misclassifications occurred between adjacent risk levels, particularly between Level 3 and Level 4. This error pattern suggests that the model’s misclassifications generally reflected minor shifts in functional severity rather than large discrepancies across non-adjacent categories. The overlap between Level 3 and Level 4 may reflect subtle similarities in gait rhythm instability, especially stride-time variability, among participants with moderate and moderately high functional risk.

### 3.9. SHAP-Based Explainability Results

**Figure 6:**
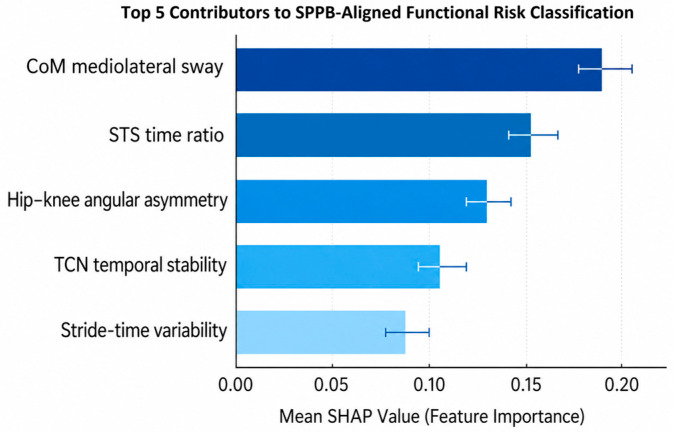
Global SHAP feature importance ranking for five-level SPPB-aligned functional risk classification.

SHAP-based feature attribution was used to examine the relative contribution of biomechanical descriptors and learned temporal features to the model’s five-level SPPB-aligned functional risk classification. The global feature importance ranking based on mean absolute SHAP values is shown in Figure 6. The five highest-ranked contributors were center-of-mass mediolateral sway, sit-to-stand time ratio, hip–knee angular asymmetry, TCN-derived temporal stability feature, and stride-time variability. These findings indicate that the proposed model did not rely solely on latent temporal representations; instead, its predictions were also influenced by biomechanically interpretable movement characteristics associated with lateral postural control, lower-limb transitional function, bilateral coordination, and gait rhythm regularity.

As shown in Figure 6, center-of-mass mediolateral sway had the largest mean absolute SHAP value among all analyzed features, suggesting that lateral postural control was the most influential contributor to the model’s classification output. The sit-to-stand time ratio was the second most important feature, indicating that delayed or inefficient transitional movement contributed meaningfully to higher functional risk classification. Hip–knee angular asymmetry and stride-time variability also showed substantial contributions, reflecting the importance of bilateral lower-limb coordination and temporal gait regularity in SPPB-aligned functional risk stratification. The presence of the TCN-derived temporal stability feature among the top-ranked contributors further suggests that learned temporal representations captured additional dynamic skeletal information beyond the handcrafted biomechanical descriptors.

**Figure 7:**
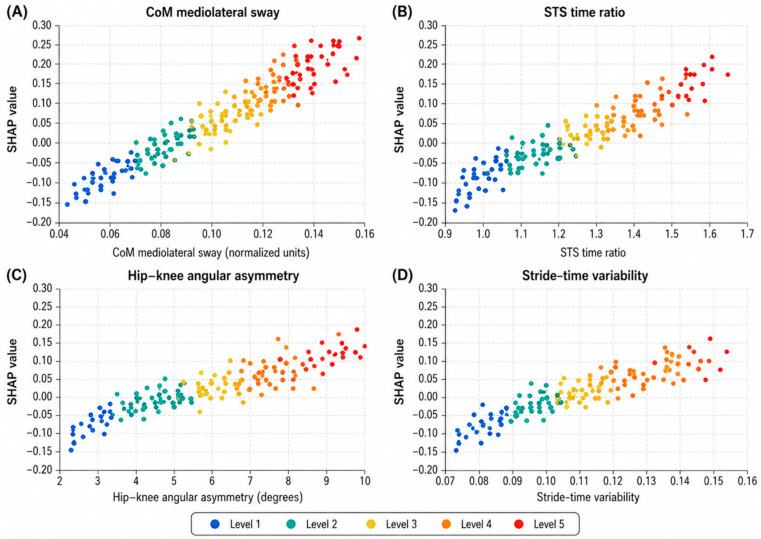
SHAP dependence plots for top-ranked handcrafted biomechanical descriptors. (**A**) center-of-mass mediolateral sway, (**B**) sit-to-stand time ratio, (**C**) hip–knee angular asymmetry, and (**D**) stride-time variability. The plots were used for post hoc interpretability only and should not be interpreted as causal evidence or clinically validated decision thresholds.

Mean absolute SHAP values indicate the average contribution magnitude of each feature to the model output across the held-out participant-independent test set. To further examine how the top-ranked handcrafted biomechanical descriptors were associated with model outputs, SHAP dependence plots were generated and are shown in Figure 7. Each point represents one participant in the held-out participant-independent test set. The x-axis indicates the observed feature value, and the y-axis indicates the SHAP contribution of that feature to the model’s expected SPPB-aligned functional risk level. Points are colored according to the corresponding five-level functional risk label.

Figure 7A shows that larger center-of-mass mediolateral sway was generally associated with positive SHAP contributions toward higher expected functional risk levels, whereas lower sway values tended to contribute toward lower functional-risk classification. Figure 7B demonstrates a similar increasing pattern for the sit-to-stand time ratio, suggesting that slower or less efficient sit-to-stand performance contributed positively to higher functional risk outputs. Figure 7C shows that greater hip–knee angular asymmetry was associated with higher SHAP values, indicating that increased bilateral lower-limb movement asymmetry contributed to higher functional risk classification. Figure 7D further shows that higher stride-time variability was generally associated with positive SHAP contributions, supporting the role of gait rhythm irregularity in functional risk stratification.

Overall, the SHAP ranking in Figure 6 and the dependence patterns in Figure 7 are consistent with the biomechanical trends reported in Table 6. Higher mediolateral sway, prolonged sit-to-stand performance, greater hip–knee asymmetry, and increased stride-time variability were all associated with higher SPPB-aligned functional risk levels. However, these analyses should be interpreted as post hoc model explanations rather than causal evidence. The SHAP values describe how each feature contributed to the trained model’s output in this dataset; they do not establish clinical causality or validated diagnostic thresholds. Therefore, Figure 6 and Figure 7 support model transparency and biomechanical plausibility, but they should not be interpreted as defining clinically actionable cut points for prospective fall-event prediction or clinical decision-making.

### 3.10. Computational Efficiency and Inference-Latency Assessment

The computational efficiency of the proposed TCAI-FallNet model was evaluated to determine whether the architecture could provide a favorable balance between model compactness, temporal modeling capacity, and SPPB-aligned functional risk classification performance. As shown in Table 7, the proposed model contained approximately 1.28 million trainable parameters. This parameter count was lower than that of the ConvLSTM baseline (1.47 million parameters) and the Transformer baseline (2.21 million parameters), while TCAI-FallNet achieved higher macro-F1, macro-AUC, and overall accuracy than both models.

The comparison with lightweight temporal baselines further contextualized the computational profile of the proposed architecture. The DS-TCN model had the smallest parameter count (0.56 million parameters), but its classification performance was lower than that of TCAI-FallNet. The TCN-only model contained 0.92 million parameters and achieved a macro-AUC of 0.88, indicating that temporal convolutional modeling captured meaningful skeletal sequence information. The MS-TCN-lite model achieved stronger performance than TCN-only and DS-TCN, with a macro-AUC of 0.89, but still remained below the proposed fused TCAI-FallNet model. These findings suggest that although TCAI-FallNet was not the smallest model in terms of parameter count, the integration of TCN-derived temporal embeddings and handcrafted biomechanical descriptors provided a stronger performance-to-complexity trade-off than the evaluated lightweight temporal alternatives.

In addition to the model-level parameter comparison, inference efficiency was assessed using the workstation environment described in the Section 2, consisting of an Intel i5-9500E CPU, an NVIDIA A4000 GPU, and the PyTorch 2.1 framework. Under this configuration, the final trained TCAI-FallNet model occupied 2.8 MB of storage space after serialization and achieved an average TCN inference latency of approximately 19–20 ms per input sequence. This latency measurement corresponded specifically to model-level inference after pose estimation, skeletal coordinate normalization, and biomechanical feature extraction had already been completed.

It is important to note that the reported latency did not include several components that would be present in a complete deployment pipeline. Specifically, video acquisition, BlazePose-based pose estimation, file input/output operations, user-interface rendering, report generation, and network communication were excluded from the timing measurements. Therefore, the reported latency should be interpreted as workstation-based model-level inference performance rather than end-to-end system latency or embedded-device performance.

Overall, the results indicate that TCAI-FallNet achieved efficient model-level inference with a compact computational footprint while achieving higher held-out test-set classification metrics than the evaluated classic and lightweight temporal baselines. The combination of moderate parameter count, small serialized model size, sub-20 ms model-level inference latency, and superior macro-averaged classification metrics suggests that the proposed architecture may support future near-real-time functional risk assessment applications. Nevertheless, additional optimization and validation on embedded devices, smartphones, or low-power edge-computing platforms remain necessary before drawing conclusions regarding real-world end-to-end deployment performance.

## 4. Discussion

### 4.1. Principal Findings and Study Positioning

This study proposed TCAI-FallNet, a lightweight contactless temporal convolutional framework for five-level SPPB-aligned functional fall-risk stratification in older adults using monocular RGB video. By integrating BlazePose-based skeletal trajectory extraction, pelvis-centered and height-normalized coordinate transformation, biomechanical feature engineering, and dilated temporal convolutional modeling, the proposed framework transformed standardized frontal-view video recordings into functional risk-level estimates.

The revised study positioning is important. Because prospective fall-event outcomes were not available, the model should not be interpreted as a validated predictor of future falls. Instead, the supervised labels represented five ordinal levels derived from a pre-specified SPPB-aligned functional risk index. Under participant-level data partitioning, TCAI-FallNet achieved a macro-AUC of 0.91 and an overall accuracy of 81.3% on the held-out test set. These findings suggest that the proposed framework can support contactless functional mobility risk stratification while maintaining interpretability and computational efficiency.

### 4.2. Interpretation of the SPPB-Aligned Functional Risk Index

A major methodological clarification in the revised manuscript is the construction of the five-level labels. The functional risk index was designed as an operational scoring scheme that combined SPPB-aligned task performance and vision-derived mediolateral trunk-control indicators. The SPPB-aligned components, including gait speed, five-times sit-to-stand performance, and balance-task completion, accounted for 60% of the index. The remaining 40% was derived from left and right lateral trunk deviation angles during walking, which were included to capture dynamic mediolateral trunk-control characteristics not directly quantified by conventional SPPB scoring.

This weighting scheme should be interpreted as a pre-specified operational design rather than as a clinically calibrated fall-event probability model. The index thresholds were used to generate ordinal supervised-learning labels, not to estimate prospective fall probability. The sensitivity analysis showed that alternative weighting schemes produced broadly consistent stratification and model performance, suggesting that the main findings were not solely dependent on the original 60/40 weighting structure. Nevertheless, future prospective studies will be needed to determine whether these functional risk levels are associated with actual fall incidence or longitudinal mobility decline.

### 4.3. Model Performance, Fusion Design, and Ablation Findings

Compared with representative classic and lightweight temporal baselines, including CNN, CNN-LSTM, ConvLSTM, Transformer, TCN-only, DS-TCN, and MS-TCN-lite models, TCAI-FallNet achieved higher macro-F1, macro-recall, macro-precision, and macro-AUC values. It should be noted that many recent SOTA action-recognition and fall-detection models are optimized for acute fall-event detection, general action recognition, or public benchmark datasets rather than standardized SPPB-aligned functional risk stratification. Therefore, the present comparison focused on representative classic and lightweight temporal baselines evaluated under the same participant-level protocol. Future studies should further benchmark TCAI-FallNet against additional lightweight skeleton-based and edge-oriented temporal architectures using harmonized SPPB-aligned datasets. Recent deep learning-based fall detection research has shown the value of combining visual pose information with temporal learning strategies for fall-related sensing tasks [25]. In contrast to fall-event detection systems, the present study focused on graded functional risk stratification before fall events occur.

The ablation results further supported the design of the proposed framework. The biomechanical-only model demonstrated that handcrafted descriptors carried meaningful functional information, whereas the TCN-only model showed that learned temporal embeddings captured important sequential skeletal patterns. The full fused model achieved the best performance, indicating that biomechanical descriptors and learned temporal features provided complementary information. Performance reductions after removing skeletal normalization, temporal smoothing, class weighting, or full-sequence modeling further supported the contribution of these design components.

The confusion matrix showed that most misclassifications occurred between adjacent levels, particularly between Levels 3 and 4. This pattern suggests that classification errors generally reflected minor shifts in functional severity rather than severe misclassification across distant levels. Such overlap may reflect the continuous nature of functional decline, especially when moderate and moderately high-risk participants present similar gait rhythm instability or stride-time variability.

### 4.4. Biomechanical Interpretability and Contactless Sensing Considerations

A key strength of the proposed framework is the integration of interpretable biomechanical descriptors with learned temporal embeddings. Mediolateral center-of-mass sway, sit-to-stand time ratio, hip–knee angular asymmetry, and stride-time variability all increased across higher functional risk levels. SHAP-based post hoc attribution further indicated that model predictions were influenced by physiologically meaningful movement characteristics. This extends prior AI-assisted dynamic postural control screening research by shifting the emphasis toward computational modeling of contactless skeletal sensing signals for functional risk stratification [26].

However, SHAP analysis should not be interpreted as causal evidence or as a source of clinically validated decision thresholds. The results indicate feature contributions to model output, not direct causal mechanisms. In addition, because the sensing setup used a single frontal monocular RGB camera, the extracted skeletal features should be interpreted as standardized two-dimensional image-plane motion descriptors rather than absolute three-dimensional biomechanical measurements. Although this design improves accessibility and reduces participant burden, robustness under different lighting conditions, occlusion, camera-angle variation, and real-world environmental noise remains to be further evaluated.

### 4.5. Deployment Considerations, Limitations

The proposed model maintained a compact deployment footprint of 2.8 MB and achieved TCN inference latency of approximately 19–20 ms per input sequence under the workstation-based evaluation setting. These results support the computational efficiency of the temporal model. From a broader perspective, the framework is consistent with trends in AI-enabled sensing and elderly fall-prevention systems, where low-latency processing, local computation, and multimodal integration are increasingly emphasized [27].

Nevertheless, the current deployment evidence should be interpreted cautiously. The reported latency refers only to model-level TCN inference after pose estimation and preprocessing; it does not include video acquisition, BlazePose processing, file input/output, user-interface rendering, report generation, or network communication. End-to-end validation on embedded devices, smartphones, or low-power edge hardware remains necessary.

Several limitations should be acknowledged. First, the labels were derived from an SPPB-aligned functional risk index rather than prospective fall-event outcomes. Second, the weighting scheme used to construct the functional risk index was operationally defined and requires future validation against longitudinal fall events or mobility decline. Third, the dataset was obtained from a single cohort of community-dwelling older adults in Taiwan, and external validation across different countries, care settings, and health conditions is required. Finally, the current study used two-dimensional frontal-view skeletal features; future work may incorporate multi-view RGB, depth sensing, inertial sensors, or ambient sensing data to improve robustness and generalizability.

## 5. Conclusions

This study presented TCAI-FallNet, a lightweight and interpretable temporal convolutional framework for contactless five-level SPPB-aligned functional fall-risk stratification in older adults using monocular RGB video. By integrating BlazePose-based skeletal trajectory extraction, pelvis-centered and height-normalized coordinate transformation, biomechanical feature engineering, and dilated temporal convolutional modeling, the proposed framework transformed standardized frontal-view video recordings into functional risk-level estimates.

In the revised study design, the supervised labels were explicitly defined as five ordinal levels derived from a pre-specified SPPB-aligned functional risk index rather than from prospective fall-event outcomes. Therefore, the proposed model should be interpreted as a classifier of SPPB-aligned functional risk levels, not as a clinically validated predictor of future falls. Under participant-level data partitioning, TCAI-FallNet achieved a macro-AUC of 0.91 and an overall accuracy of 81.3% on the held-out test set. Ablation analyses further indicated that biomechanical descriptors and TCN-derived temporal embeddings provided complementary information, supporting the value of the proposed hybrid fusion design.

The SHAP-based post hoc analysis suggested that model predictions were influenced by physiologically meaningful movement characteristics, including mediolateral center-of-mass sway, sit-to-stand time ratio, hip–knee angular asymmetry, and stride-time variability. In addition, the model maintained a compact size of 2.8 MB and achieved TCN inference latency of approximately 19–20 ms per input sequence under workstation-based evaluation. These findings support the computational efficiency of the proposed temporal model, although they should not be interpreted as full end-to-end edge–device validation.

Overall, TCAI-FallNet provides a feasible contactless sensing framework for SPPB-aligned functional mobility risk stratification in community-dwelling older adults. Future research should validate the proposed functional risk levels against prospective fall events and longitudinal mobility decline, examine generalizability across external cohorts and care settings, evaluate end-to-end performance on embedded or mobile edge-computing platforms, report confidence intervals for AUC and macro-F1, conduct repeated cross-validation or external validation, and perform formal statistical comparisons with additional lightweight skeleton-based models.

## Figures and Tables

**Table 1:** Components of the SPPB-aligned functional risk index.

Component	Input indicator	Maximum Contribution	Rationale
Gait speed	3 m usual-pace walking speed	30	Core mobility indicator reflecting walking efficiency and functional mobility
Five-times sit-to-stand	Completion time or inability to complete 5× STS	15	Indicator of lower-limb strength and transitional movement ability
Balance performance	Completion of static balance tasks	15	Indicator of static postural control
Left lateral trunk deviation angle	Leftward trunk lean relative to the core midline during walking	20	Vision-derived indicator of mediolateral trunk control
Right lateral trunk deviation angle	Rightward trunk lean relative to the core midline during walking	20	Vision-derived indicator of mediolateral trunk control
Total	—	100	Higher scores indicate greater SPPB-aligned functional risk burden

**Table 2:** Mapping between the SPPB-aligned functional risk index and five-level labels.

Functional Risk Index (%)	Assigned Level	Label Interpretation
≤5%	Level 1	Low functional risk
>5% to ≤17.5%	Level 2	Slight functional risk
>17.5% to ≤30%	Level 3	Moderate functional risk
>30% to ≤50%	Level 4	Moderately high functional risk
>50%	Level 5	High functional risk

**Table 3:** Class distribution of the SPPB-aligned functional risk levels across data subsets.

Functional Risk Level	Functional Risk Index Range	Full Cohort Numbers (%)	Training Numbers (%)	Validation Numbers (%)	Test Numbers (%)
Level 1	≤5%	128 (18.6%)	77 (60.2%)	25 (19.5%)	26 (20.3%)
Level 2	>5% to ≤17.5%	146 (21.2%)	88 (60.3%)	29 (19.7%)	29 (20.0%)
Level 3	>17.5% to ≤30%	122 (17.7%)	73 (59.8%)	24 (19.7%)	25 (20.5%)
Level 4	>30% to ≤50%	151 (22.0%)	91 (60.3%)	30 (19.9%)	30 (20.8%)
Level 5	>50%	141 (20.5%)	83 (58.8%)	29 (20.6%)	29 (20.6%)
Total	—	688 (100%)	412 (59.9%)	137 (19.9%)	139 (20.2%)

**Table 4:** Input features and training settings for baseline and ablation models.

Model	Normalized Skeletal Sequence	Biomechanical Descriptors	Feature Fusion	Class Weighting	Same Participant-Level Split	Purpose
CNN	Yes	No	No	Yes	Yes	Spatial–temporal baseline
CNN-LSTM	Yes	No	No	Yes	Yes	Recurrent sequence baseline
ConvLSTM	Yes	No	No	Yes	Yes	Convolutional recurrent baseline
Transformer	Yes	No	No	Yes	Yes	Attention-based sequence baseline
Biomechanical-only classifier	No	Yes	No	Yes	Yes	Handcrafted-feature ablation
TCN-only	Yes	No	No	Yes	Yes	Learned temporal embedding ablation
DS-TCN	Yes	No	No	Yes	Yes	Depthwise-separable lightweight temporal baseline
MS-TCN-lite	Yes	No	No	Yes	Yes	Multi-scale lightweight temporal baseline
Proposed TCAI-FallNet	Yes	Yes	Yes	Yes	Yes	Full hybrid model

**Table 5:** Dataset summary and sequence characteristics.

Parameter	Mean ± SD	Range
Participants	688	-
Female participants	442 (64.2%)	-
Age (years)	74.3 ± 6.2	65–92
Standing sequence length (frames)	309 ± 12	300–330
STS sequence length (frames)	182 ± 25	140–240
Gait sequence length (frames)	210 ± 33	150–270

**Table 6:** Biomechanical feature differences across SPPB-aligned functional risk levels.

Feature	Level 1 Mean ± SD	Level 2 Mean ± SD	Level 3 Mean ± SD	Level 4 Mean ± SD	Level 5 Mean ± SD	*p*-Value
CoM ML Sway (normalized units)	0.062 ± 0.018	0.071 ± 0.021	0.084 ± 0.024	0.102 ± 0.028	0.118 ± 0.033	<0.001
STS Ratio	1.12 ± 0.21	1.18 ± 0.24	1.25 ± 0.28	1.37 ± 0.32	1.49 ± 0.38	<0.001
Hip–Knee asymmetry (degrees)	3.8 ± 1.4	4.5 ± 1.6	5.8 ± 1.9	6.9 ± 2.3	7.3 ± 2.5	<0.001
Stride-time variability	0.084 ± 0.025	0.092 ± 0.027	0.107 ± 0.032	0.122 ± 0.038	0.134 ± 0.041	<0.001

**Table 7:** Model-level comparison of TCAI-FallNet with representative classic and lightweight temporal baselines.

Model	Parameters	Macro-F1	Macro-Precision	Macro-Recall	Macro-AUC	Accuracy
CNN	0.74 M	0.71	0.74	0.71	0.83	70.5%
CNN-LSTM	1.05 M	0.73	0.75	0.71	0.84	72.7%
ConvLSTM	1.47 M	0.76	0.78	0.75	0.86	75.5%
Transformer	2.21 M	0.77	0.79	0.76	0.87	76.3%
TCN-only	0.92 M	0.78	0.80	0.77	0.88	77.7%
DS-TCN	0.56 M	0.75	0.78	0.75	0.86	76.0%
MS-TCN-lite	1.08 M	0.79	0.81	0.78	0.89	79.1%
Proposed TCAI-FallNet	1.28 M	0.82	0.84	0.81	0.91	81.3%

**Table 8:** Ablation analysis of TCAI-FallNet components.

Model Variant	Biomechanical Descriptors	TCN Embeddings	Normalization	Temporal Smoothing	Class Weighting	Macro-F1	Macro-AUC	Accuracy
Biomechanical-only classifier	Yes	No	—	—	Yes	0.68	0.80	68.3%
TCN-only	No	Yes	Yes	Yes	Yes	0.78	0.88	77.7%
TCAI-FallNet without normalization	Yes	Yes	No	Yes	Yes	0.76	0.86	76.3%
TCAI-FallNet without temporal smoothing	Yes	Yes	Yes	No	Yes	0.79	0.89	79.1%
TCAI-FallNet without class weighting	Yes	Yes	Yes	Yes	No	0.77	0.88	78.4%
Task-wise segmented input	Yes	Yes	Yes	Yes	Yes	0.79	0.89	78.4%
Proposed TCAI-FallNet	Yes	Yes	Yes	Yes	Yes	0.82	0.91	81.3%

**Table 9:** Sensitivity analysis of alternative functional risk index weighting schemes.

Weighting Scheme	Gait Speed	5× STS	Balance	Left Lateral Trunk Deviation	Right Lateral Trunk Deviation	Agreement with Original Labels	Weighted kappa	Macro-F1	Macro-AUC
Proposed operational scheme	30	15	15	20	20	Reference	Reference	0.82	0.91
Equal-weight scheme	20	20	20	20	20	88.2%	0.84	0.80	0.90
SPPB-dominant scheme	35	20	20	12.5	12.5	86.9%	0.82	0.79	0.89

## Data Availability

The data presented in this study are available upon request from the corresponding author. The raw video and skeletal keypoint data are not publicly available due to privacy and ethical restrictions involving human participants, as well as proprietary considerations.

## Abbreviations

The following abbreviations are used in this manuscript:

AI	Artificial Intelligence
AUC	Area Under the Receiver Operating Characteristic Curve
CNN	Convolutional Neural Network
CoM	Center of Mass
ConvLSTM	Convolutional Long Short-Term Memory
FN	False Negative
FP	False Positive
fps	Frames per Second
GPU	Graphics Processing Unit
IMU	Inertial Measurement Unit
IoT	Internet of Things
IRB	Institutional Review Board
LSTM	Long Short-Term Memory
ML	Mediolateral
ReLU	Rectified Linear Unit
ROC	Receiver Operating Characteristic
RGB	Red–Green–Blue
SHAP	SHapley Additive exPlanations
SPPB	Short Physical Performance Battery
STS	Sit-to-Stand
STV	Stride-Time Variability
TCAI-FallNet	Temporal Convolutional AI Fall-Risk Network
TCN	Temporal Convolutional Network
TP	True Positive
WHO	World Health Organization
